# Supplementary material for: The Effects of External Cue Overlap and Internal Goals on Selective Memory Retrieval as Revealed by Electroencephalographic (EEG) Neural Pattern Reinstatement
Source: Eur J Neurosci. 2025 Jul 13;62(1):e70194. doi: 10.1111/ejn.70194 (PMC12256160; doi:10.1111/ejn.70194)
Supplement: Supplementary file 1 — Table S1 Mean and range number of training time‐bins contributing to the reactivation scores across all conditions and test time‐bins in each test time‐window (early time‐window: 200–500 ms, and late time‐window: 500–800 ms) and experiment. Table S2. LMM on the Reactivation Scores with Item Type (Targets/Non‐targets), Target Designation (Target‐Audio/Target‐Picture), Test Time‐Window (Early: 200–500‐ms/Late: 500–800‐ms), and Experiment (Experiment‐1/Experiment‐2) as Fixed Effects and Subjects as Random Intercepts. Table S3. LMM model parameters split by Experiment. Table S4. LMM on the Reactivation Scores with Item Type (Targets/Non‐targets) and Cue‐Target Overlap (High/Low) as Fixed Effects and Subjects as Random Intercepts during the Late Test Window (T2, 500–800 ms). [file EJN-62-0-s003.docx]

# **S1. Supplementary Methods and Results**

**S1.1 Study phase decoding**

In an initial step we used linear discriminant analysis (LDA) to decode study phase neural patterns when participants saw object pictures versus heard object names. The goal of this analysis was to inform selection of time windows for the study-test decoding between 0 and 1,000 ms post stimulus. We trained the LDA classifier to discriminate between picture and audio trials by iteratively training on all trials but one and testing on the left-out trial (a leave-one-out procedure; Linde-Domingo et al., 2019). Classifier features were ERP amplitudes from the 64 scalp electrodes, after subtraction of a 200 ms pre-stimulus baseline. Downsampling was applied to create approximately 8 ms time bins by averaging across time-points, temporal smoothing and multivariate noise normalization were applied as in the main study-test analysis. Trials were sub-sampled and mean classifier fidelity value determined over 12 iterations (see Methods, Multivariate decoding analyses in main manuscript for details).

We found that the LDA classifier could confidently assign study phase neural patterns to picture and auditory trials throughout almost the entire study phase time window in both experiments (Figure S1). One-sample *t*-tests on the classifier fidelity values in each encoding time-bin showed significant and reliable above zero decoding from ~ 50 ms until ~ 1,000 ms after stimulus onset in Experiment 1 (all *p* $\leq$ .001, with exception of time-bin four: 24-32 ms, *p* $\leq$ .05, and time-bin seven: 48-56 ms, *p* $\leq$ .01, corrected with the Benjamini-Hochberg method; Benjamini & Hochberg, 1995) and from ~ 30 until ~ 1,000 ms in Experiment 2, after correcting for multiple comparisons (all *p*-values $\leq$ .001, with exception of time-bins 4 to 8: 24-64 ms, *p* $\leq$ .05, and time-bin 9: 64-72 ms, *p* $\leq$ .01, adjusted with the Benjiamini-Hochberg correction). As these tests were run on all encoding time-bins, used the Benjiamini-Hochberg False Discovery Rate (FDR) correction to maximise sensitivity, while minimising the chance of incurring in Type I errors when running multiple comparisons. We therefore used the study phase data from ~ 150 to 1,000 as training for the classifiers testing test phase reinstatement.

**S1.2 Test phase decoding**

*S1.2.1 Decoding study-test reinstatement during memory retrieval.*

We controlled for perceptual similarity with test cues when quantifying memory-related study-test reinstatement for targeted and non-targeted items in each test block. We did this by subtracting the mean of the unstudied new item classifier fidelity scores from the mean target and non-target classifier fidelities per participant and test block, therefore shifting the classifier boundary orthogonal to the decision hyperplane for each test block (Figure S2). This adjusted classifier fidelity can be interpreted in terms of neural reinstatement for studied items *relative* to new (unstudied) items, which in turn do not elicit memory retrieval.

*S1.2.2 Study time-bin selection for computing reactivation scores during memory retrieval*

Figure S3 illustrates the most discriminative (top) training bins at the group-level study-test reinstatement maps for each condition (Figure 2). These were selected to compute the reactivation scores for each condition (see 3.5.3 in the main manuscript for details). Table S1 summarises the number, mean, and range of study bins selected across conditions and test time-bins in each test time-window of interest (early: 200-500 ms, and late: 500-800 ms).

Table S1. Mean and range number of training time-bins contributing to the reactivation scores across all conditions and test time-bins in each test time-window (early time-window: 200-500 ms, and late time-window: 500-800 ms) and experiment.

|  | | Experiment 1 | | | | Experiment 2 | | | |
| --- | --- | --- | --- | --- | --- | --- | --- | --- | --- |
| Test-Window | | *n* | Mean | Min | Max | *n* | Mean | Min | Max |
| Early | 200-500 ms | 38 | 54.17 | 40.00 | 68.00 | 37 | 50.98 | 33.50 | 68.00 |
| Late | 500-800 ms | 38 | 54.82 | 40.00 | 63.50 | 38 | 56.90 | 50.50 | 62.25 |

Note: *n* is the number of 8-ms test time-bins that show test phase reactivation after averaging the outputs of classifiers trained in the top-ranked study phase bins in each test time-window (early: 200-500 ms, late: 500-800 ms).

*S1.2.2 Decoding goal-related neural patterns during retrieval attempts.*

To assess goal-related reinstatement during the recollection time-window, we applied LDA classifiers trained on the study phase (pictures/ auditory words) to the test phase data from new item trials. Since no information should be retrieved on new trials, any difference in reinstated neural patterns can be assumed to index differences in retrieval goals (Rugg & Wilding, 2000). Here, retrieval goals had been manipulated in the Target-Picture block versus the Target-Audio block.

However, in both experiments the LDA classifier was unable to reliably distinguish at test between attempting to retrieve picture targets and attempting to retrieve audio targets. In Experiment 1, the classifier fidelity measure revealed two non-significant clusters of reinstatement from approx. 400 to 800 ms after the retrieval cue (cluster *p* = .202, .422). In Experiment 2, a slightly later-onsetting cluster was found between approximately 600 to 800 ms that was not statistically significant (cluster *p* = .080).

**S1.4 Supplementary results: Reactivation scores during memory retrieval**

The following tables show the full model results of the linear mixed effect model (LMMs) run on the reactivation scores (see 3.6 in the main manuscript for details).

| Table S2. *LMM on the Reactivation Scores with Item Type (Targets/Non-targets), Target Designation (Target-Audio/Target-Picture), Test Time-Window (Early: 200-500-ms/Late: 500-800-ms), and Experiment (Experiment-1/Experiment-2) as Fixed Effects and Subjects as Random Intercepts.* | | | | |
| --- | --- | --- | --- | --- |
| Model Terms | *dfs* | *F* | *p* | *η_p_^2^* |
| Item Type | 1,378 | 0.30 | .585 | 0.001 |
| Target Designation | 1, 378 | 0.11 | .738 | <0.001 |
| Test Time-Window | 1, 378 | 20.23 | <.001*** | 0.05 |
| Experiment | 1, 54 | 7.73 | .007** | 0.13 |
| Item Type x Target Designation | 1, 378 | 9.68 | .002** | 0.03 |
| Item Type x Test Time-Window | 1, 378 | 0.64 | .423 | 0.002 |
| Item Type x Experiment | 1, 378 | 0.08 | .773 | <.001 |
| Target Designation x Test Time-Window | 1, 378 | 0.27 | .606 | 0.001 |
| Target Designation x Experiment | 1, 378 | 5.10 | .025* | 0.01 |
| Test Time-Window x Experiment | 1, 378 | 1.37 | .243 | 0.004 |
| Item Type x Target Designation x Test Time-Window | 1, 378 | 0.10 | .752 | <.001 |
| Item Type x Target Designation x Experiment | 1, 378 | 18.66 | <.001*** | 0.05 |
| Item Type x Test Time-Window x Experiment | 1, 378 | 1.75 | .187 | 0.01 |
| Target Designation x Test Time-Window x Experiment | 1, 378 | 2.88 | .090 | 0.01 |
| Item Type x Target Designation x Test Time-Window x Experiment | 1, 378 | 4.29 | .039* | 0.01 |

*Note*: * p < .05, ** p < .01, *** p < .001

| Table S3. *LMM model parameters split by Experiment.* | | | | | | | | |
| --- | --- | --- | --- | --- | --- | --- | --- | --- |
|  | Experiment 1 | | | | Experiment 2 | | | |
| *Model Terms* | *dfs* | *F* | *p* | *η_p_^2^* | *dfs* | *F* | *p* | *η_p_^2^* |
| Item Type | 1,378 | 0.35 | .555 | 0.001 | 1,378 | 0.03 | .855 | <0.001 |
| Target Designation | 1,378 | 1.85 | .175 | 0.01 | 1,378 | 3.36 | .068 | 0.01 |
| Test Time-Window | 1,378 | 5.54 | .019* | 0.01 | 1,378 | 16.06 | <.001*** | 0.04 |
| Item Type x Target Designation | 1,378 | 0.73 | .393 | 0.002 | 1,378 | 27.60 | <.001*** | 0.07 |
| Item Type x Test Time-Window | 1,378 | 0.14 | .714 | <0.001 | 1,378 | 2.25 | .134 | 0.01 |
| Target Designation x Test Time-Window | 1,378 | 0.70 | .404 | 0.002 | 1,378 | 2.45 | .118 | 0.01 |
| Item Type x Target Designation x Test Time-Window | 1,378 | 1.54 | .215 | 0.004 | 1,378 | 2.85 | .092 | 0.01 |

*Note*: * p < .05, ** p < .01, *** p < .001

S**1.5 Supplementary results: Reactivation scores and left parietal ERPs by Cue-Target Overlap**

The following tables shows the results of the LMMs run on the reactivation scores (Table S4) and the left parietal ERPs (Table S5) averaged across the two Experiments (*n* = 56) during the late test time-window from 500-800 ms (see 3.6 in the main manuscript for details).

| Table S4. *LMM on the Reactivation Scores with Item Type (Targets/Non-targets) and Cue-Target Overlap (High/Low) as Fixed Effects and Subjects as Random Intercepts during the Late Test Window (T2, 500-800 ms).* | | | | |
| --- | --- | --- | --- | --- |
| Model Terms | *dfs* | *F* | *p* | *η_p_^2^* |
| Item Type | 1,165 | 0.60 | .439 | 0.004 |
| Overlap | 1,165 | 5.19 | .024* | 0.03 |
| Item Type x Overlap | 1,165 | 13.50 | <.001*** | 0.08 |

*Note*: * p < .05, ** p < .01, *** p < .001

| Table S5. *LMM on the Left Parietal ERP Effects with Item Type (Targets/Non-targets), Cue-Target Overlap (High/Low) as Fixed Effects and Subjects as Random Intercepts during the Late Test Window (T2, 500-800 ms).* | | | | |
| --- | --- | --- | --- | --- |
| Model Terms | *dfs* | *F* | *p* | *η_p_^2^* |
| Item Type | 1,165 | 14.50 | <.001*** | 0.08 |
| Overlap | 1,165 | 2.10 | .149 | 0.01 |
| Item Type x Overlap | 1,165 | 21.30 | <.001*** | 0.11 |

*Note*: * p < .05, ** p < .01, *** p < .001

S**1.6 Supplementary analysis of Memory Reactivation by Study Format and External Cue Overlap**

The main aim of this study was to investigate whether reinstatement is target-selective by comparing the amount of target versus non-target reinstatement within task blocks with varying retrieval goals and cues. However, we can also ask a slightly different, complementary question: is information about a given studied source reinstated more under a retrieval goal that prioritises that source than under a retrieval goal that prioritises a different source. To test this, we needed to compare the amount of reinstatement *between* blocks, when items were studied in the same format under different retrieval goals. For example, we compared reinstatement of study phase information about items studied as audios when they were targets (in the target-audio block) versus when they were non-targets (in the target-picture block). We obtained reactivation scores in the same way as for the main analysis, focusing on the late test window (500-800 ms) where the principal reinstatement effects were found (section 4.2.1 and Figure 2).

We ran separate analyses on the reactivation scores for auditory words and for pictures to assess whether the different test cues in the two experiments impacted how selectively people retrieved each item format across test blocks. Each LMM had fixed effects of Item Type (targets/non-targets) and Experiment (experiment 1: retrieval word cues/retrieval picture cues), and subjects as random intercepts. For auditory reactivation this showed non-significant main effects of Item Type *F*(1,54) = 0.78, p = .380, *η_p_^2^* = 0.01 and Experiment, *F*(1, 54) = 0.45, *p* = .507, *η_p_^2^* = 0.01, but a marginal yet borderline significant Item Type x Experiment interaction *F*(1, 54) = 4.03, *p* = .050, *η_p_^2^* = 0.07. The interaction showed that the differences in reactivation between auditory targets in the two experiments and auditory non-targets in the two experiments were numerically different, despite the pairwise comparisons were not significant. *Post hoc* tests showed that the reactivation scores for targeted auditory words were numerically but not significantly greater in Experiment 2 (where retrieval cues were line drawings, *M* = 0.15) than in Experiment 1 (where retrieval cues were visual words, *M* = 0.08 in Experiment 1), *t*(108) = -0.94, *p* = .351, Cohen’s *d* = 0.18. *Non*-targeted auditory words were instead numerically but not significantly reactivated more strongly in Experiment 1 (*M* = 0.14) than in Experiment 2 (*M* = 0.003), *t*(108) = 1.89, *p* = .062, Cohen’s *d* = 0.36. Despite the pairwise differences in the amount of auditory reactivation did not significantly vary with retrieval goals (i.e., target designation), we detected significant reinstatement separately for auditory non-targets in Experiment 1 but not for auditory non-targets in Experiment 2, for and auditory targets in Experiment 2 but not auditory targets in Experiment 1 (see Figure 2 and section 4.2.1 in the main manuscript). Thus, format-specific auditory reinstatement followed the external overlap with the retrieval cues at least for items that were non-targeted.

For picture reactivation we found a strong effect of external cue overlap. The model showed a non-significant main effect of Item Type *F*(1,54) = 0.09, *p* = .766, *η_p_^2^* = 0.002, and a non-significant interaction, *F*(1, 54) = 3.08, *p* = .085, *η_p_^2^* = 0.05, but a significant main effect of Experiment, *F*(1,54) = 13.12, *p* = .001, *η_p_^2^* = 0.20, showing that reactivation scores for items studied as pictures was overall greater in Experiment 2 (M = 0.27) than in Experiment 1 (M = 0.05), when the retrieval cues overlapped strongly with pictures. Thus, the results showed that format-specific neural reactivation during retrieval closely tracked the overlap between retrieval cues and the studied source. Irrespective of retrieval goals (i.e., the target designation), reinstatement was only present and slightly greater for non-targets studied as auditory words in Experiment 1 (when retrieval cues were visual words) and for items studied as pictures in Experiment 2 (when retrieval cues were line drawings).

In a second analysis we then collapsed the trials averaging across experiments, coding according to the external overlap between retrieval cues and the studied source. Since this analysis was organised by format not by retrieval block, high (external) overlap items were trials (both targets and non-targets) that were studied as auditory words in Experiment 1 and those that were studied as pictures in Experiment 2, whereas low (external) overlap items were trials that were studied as pictures in Experiment 1 and auditory words in Experiment 2. Importantly, this analysis ensured that the same combination of classifiers (study bins used to decode auditory words versus pictures) contributed to decoding performance each overlap condition. A LMM on these collapsed reactivation scores had fixed effects of Item Type (targets/non-targets) and Cue Overlap (high/low) and participants as random intercepts. This revealed a non-significant main effect of Item Type *F*(1, 165) = 0.60, *p* < .439, *η_p_^2^* = 0.004, but a significant main effect of and Cue Overlap, *F*(1, 165) = 13.54, *p* < .001, *η_p_^2^* = 0.08. These were qualified by a significant interaction, *F*(1, 165) = 5.19, *p* = .024, *η_p_^2^* = 0.03. *Post hoc* pairwise comparisons converged with those in the main analysis. These showed that for targets, the difference in reinstatement was numerically (but not significantly) greater in the high (*M* = 0.16) than the low (*M* = 0.12) cue overlap conditions, *t(*165) = 0.992, *p* = .323, Cohen’s *d* = 0.08, while for non-targets, neural reactivation was significantly greater for items with high (*M* = 0.22) than low (*M* = 0.01) cue overlap, *t*(165) = 4.21, *p* < .001, Cohen’s *d* = 0.33.

S2. Reference

Bates, D., Maechler, M., Bolker, B., Walker, S. (2015). Fitting Linear Mixed-Effects Models Using lme4. Journal of Statistical Software, 67(1), 1-48. doi:10.18637/jss.v067.i01.

Benjamini, Y., & Hochberg, Y. (1995). Controlling the False Discovery Rate: A Practical and Powerful Approach to Multiple Testing. *Journal of the Royal Statistical Society: Series B (Methodological)*, *57*(1), 289–300. https://doi.org/10.1111/j.2517-6161.1995.tb02031.x

Linde-Domingo, J., Treder, M. S., Kerrén, C., & Wimber, M. (2019). Evidence that neural information flow is reversed between object perception and object reconstruction from memory. *Nature Communications*, *10*(1). <https://doi.org/10.1038/s41467-018-08080-2>

Moccia, A., & Morcom, A. M. (2021). Cue overlap supports preretrieval selection in episodic memory: ERP evidence. *Cognitive, Affective, & Behavioral Neuroscience*. https://doi.org/10.3758/s13415-021-00971-0

Morey, R. D. (2008). Confidence Intervals from Normalized Data: A correction to Cousineau (2005). Tutorials in Quantitative Methods for Psychology, 4(2), 61–64. https://doi. org/10.20982/tqmp.04.2.p061
