# Supplementary figures and images for: The Effects of External Cue Overlap and Internal Goals on Selective Memory Retrieval as Revealed by Electroencephalographic (EEG) Neural Pattern Reinstatement
Source: Eur J Neurosci. 2025 Jul 13;62(1):e70194. doi: 10.1111/ejn.70194 (PMC12256160; doi:10.1111/ejn.70194)

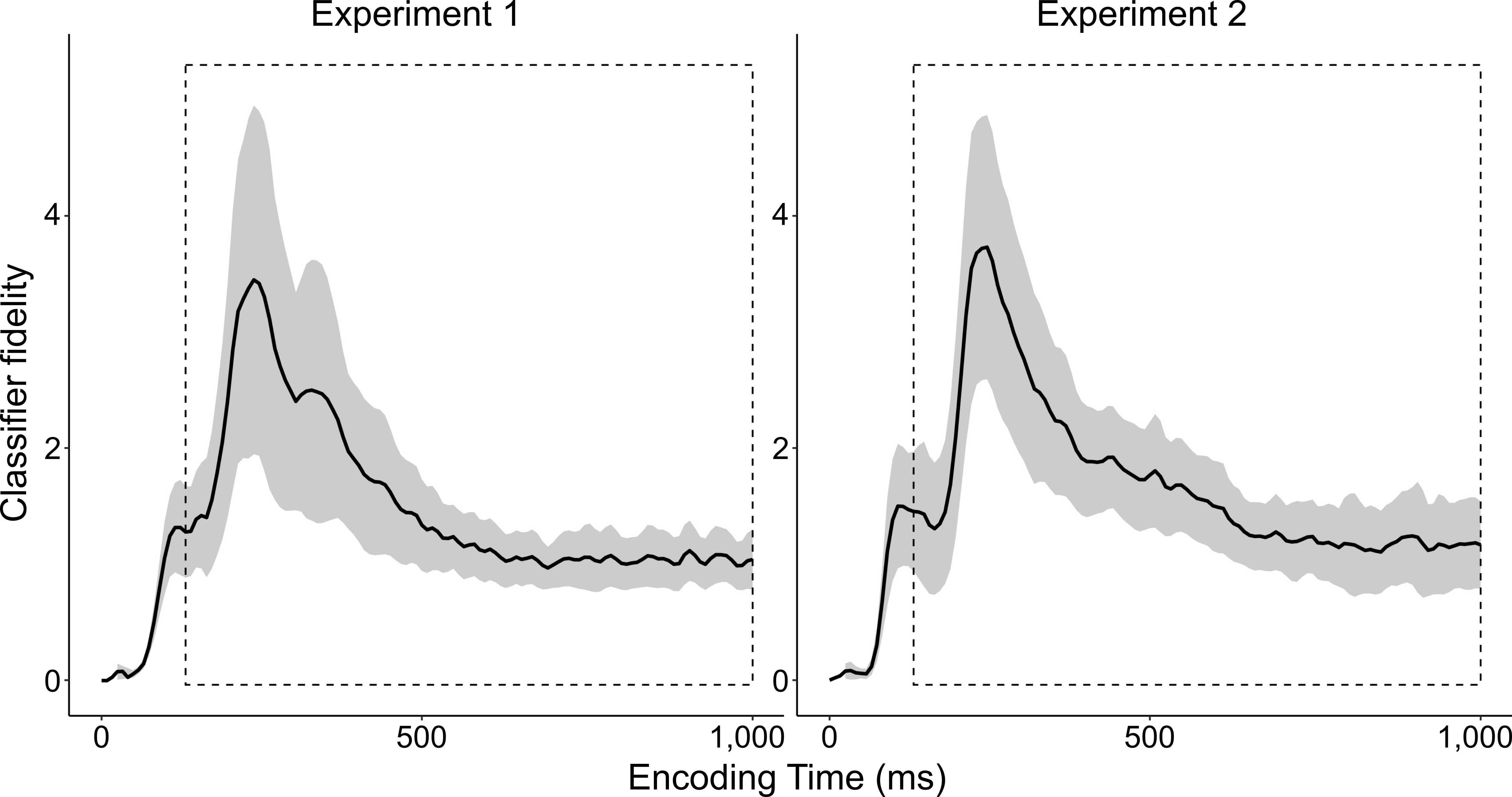

Supplement: Supplementary file 2 — Figure S1 Mean classifier fidelity of study phase neural patterns in Experiment 1 and Experiment 2. The shaded area represents the 95% confidence intervals around the encoding time‐bins showing significant above‐zero decoding fidelity (see text for details). Dashed lines are the study time window (~150–1000 ms) used as training for the test phase decoding analyses. [file EJN-62-0-s004.tiff]

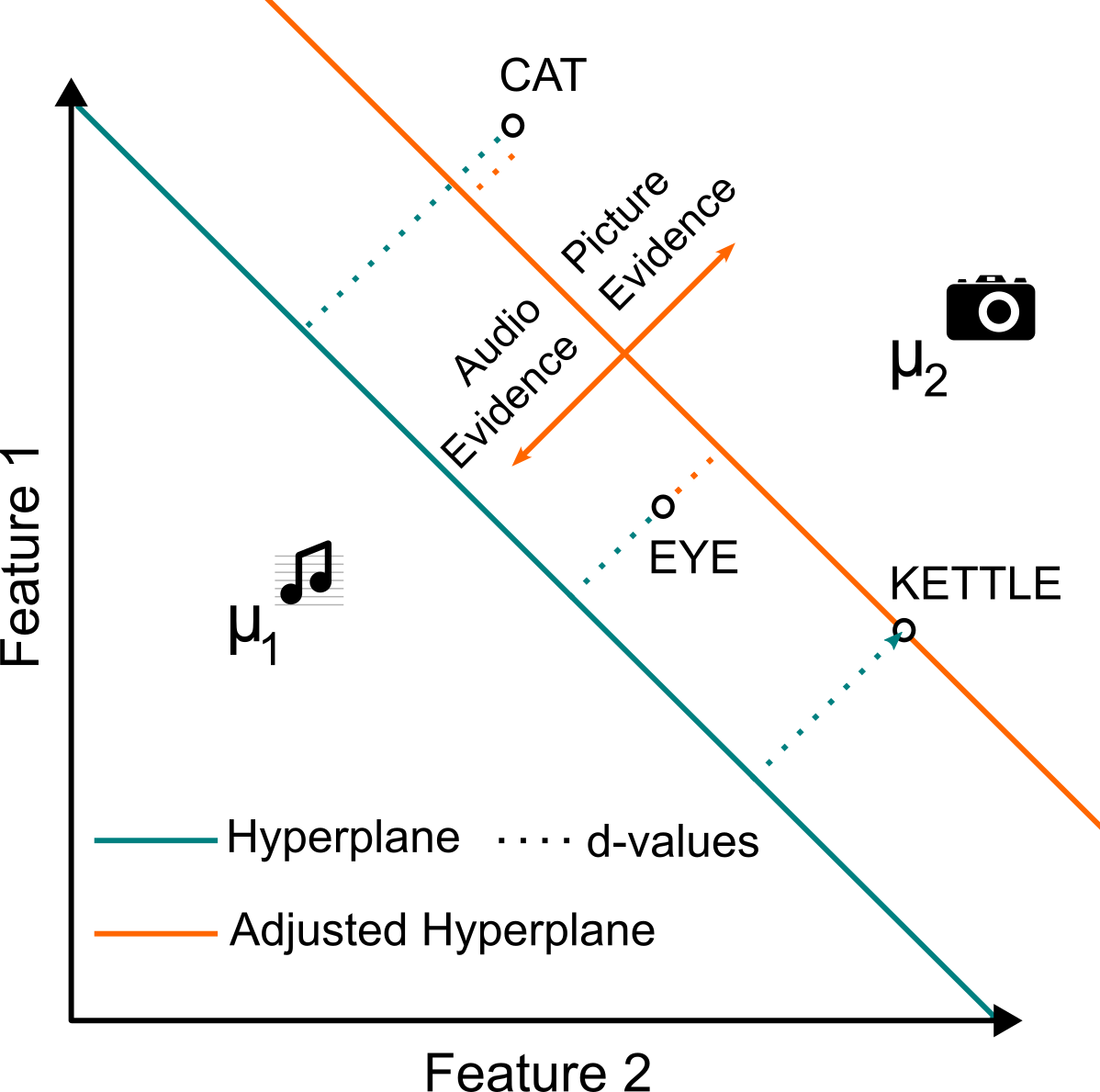

Supplement: Supplementary file 3 — Figure S2 Linear discriminant classification analyses. Images illustrate the multivariate classification approach used on test trials after training LDA classifiers to classify neural patterns associated with hearing words and seeing pictures during encoding, and the adjustment for new (unstudied) items. The position of items in relation to the x‐ and y‐axes illustrate (for a 2D feature space) the position of trained LDA classification boundaries (hyperplanes) and test items within the multivariate coordinate spaces for the test data. The music icons denote the position of the auditory word study conditions relative to the LDA hyperplane after training (with means μ1) and the camera icons the position of the picture study conditions (with means μ2). The teal lines show the hyperplanes determined by LDA on the training (study phase) data. The orange lines show the adjusted hyperplane after d‐values for unstudied items were taken into account for each block (see text for further details). The dotted lines show the distance to the hyperplane (d‐values). Teal dotted lines are the original d‐values before new items adjustment, whereas the orange dotted lines are the adjusted d‐values and therefore represent the classifier evidence for audio (negative values) and picture (positive value) mnemonic information, respectively. [file EJN-62-0-s001.tiff]

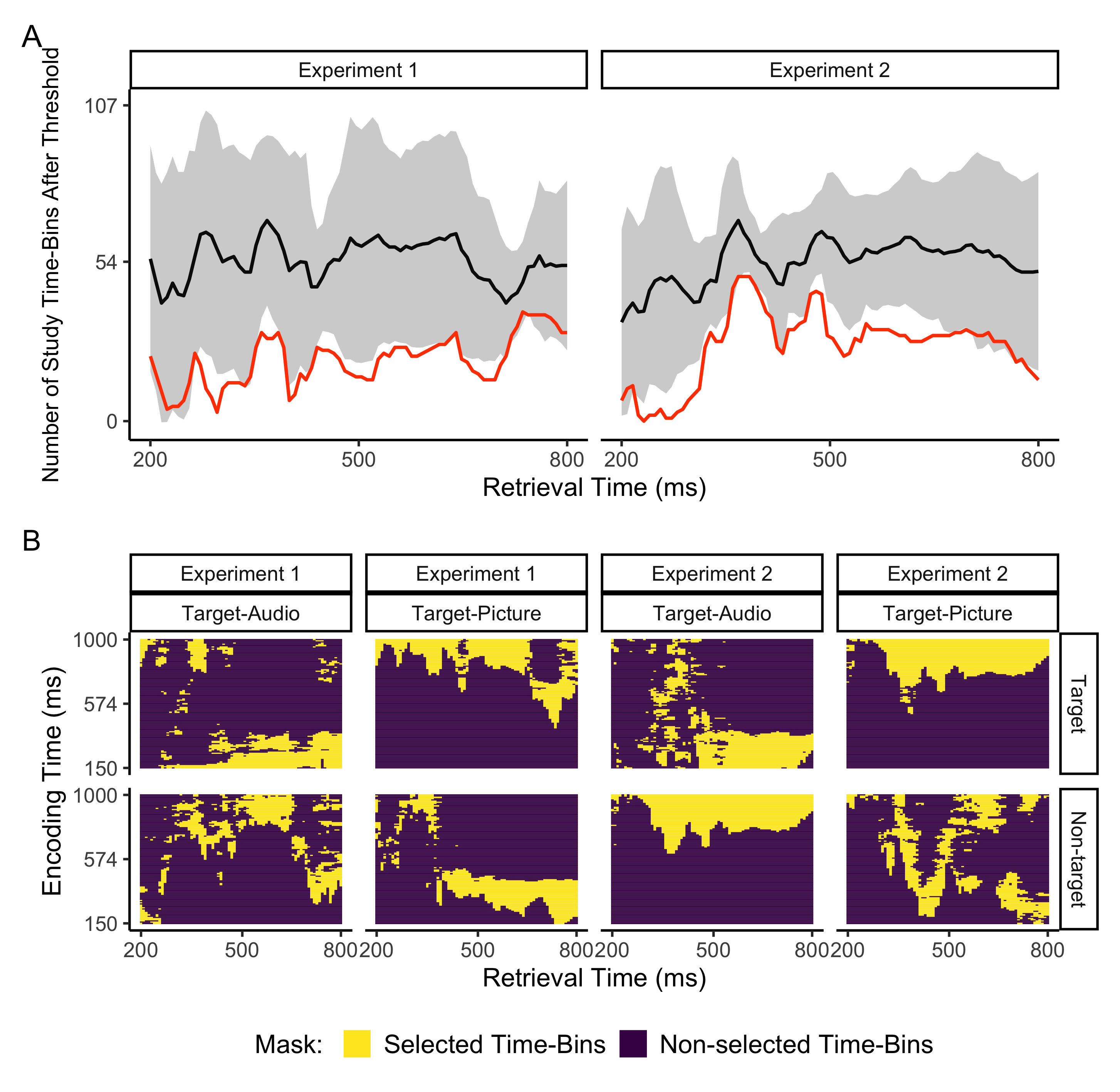

Supplement: Supplementary file 4 — Figure S3 Training time bin selection method for computing memory reactivation index. A) Number of encoding time‐bins showing reliable classifier fidelity (positive d‐values) in at least 50% of the sample for each 8‐ms time point at retrieval across experimental conditions (i.e., targets and non‐targets in the target‐audio and target‐picture test blocks) and separately for each experiment. Black lines show the means across conditions and the shaded areas are the within‐subject 95% confidence intervals. Red lines are the minimum number of time bins that survive the threshold across conditions. This minimum number was used to determine how many top bins to select for decoding the test phase data. Top bins were those that contributed the most (from most to least positive d‐values) to the group‐level fidelity for each condition (see Section 2.5.3 in the main manuscript). B) Heatmaps show the top‐ranked encoding time‐bins that contribute the most to the group‐level classifier fidelity for each retrieval time‐point, item type (Target/Non‐target), target designation (Target‐Audio/Target‐Picture) and experiment (Experiment 1/Experiment 2). Because the top‐ranking bins are selected based on the minimum number of bins surviving the directional threshold across conditions, this selection procedure allows that the same number but not the same encoding bins are selected across LDA classes. [file EJN-62-0-s002.tiff]
